# Silver-pig skin nanocomposites and mesenchymal stem cells: suitable antibiofilm cellular dressings for wound healing

**DOI:** 10.1186/s12951-017-0331-0

**Published:** 2018-01-10

**Authors:** Mario Alberto Pérez-Díaz, Phaedra Silva-Bermudez, Binisa Jiménez-López, Valentín Martínez-López, Yaaziel Melgarejo-Ramírez, Ana Brena-Molina, Clemente Ibarra, Isabel Baeza, M. Esther Martínez-Pardo, M. Lourdes Reyes-Frías, Erik Márquez-Gutiérrez, Cristina Velasquillo, Gabriel Martínez-Castañon, Fidel Martinez-Gutierrez, Roberto Sánchez-Sánchez

**Affiliations:** 1 Laboratorio de Biotecnología, Instituto Nacional de Rehabilitación Luis Guillermo Ibarra Ibarra, Calz. México Xochimilco No 289 Col. Arenal de Guadalupe, C.P.14389 Mexico City, Mexico; 2 Unidad de Ingeniería de Tejidos, Terapia Celular y Medicina Regenerativa, Instituto Nacional de Rehabilitación Luis Guillermo Ibarra Ibarra, Calz. México Xochimilco No 289 Col. Arenal de Guadalupe, C.P.14389 Mexico City, Mexico; 3 Laboratorio de Biomembranas, Escuela Nacional de Ciencias Biológicas, Instituto Politécnico Nacional, Prolongación de Carpio y Plan de Ayala s/n, Col. Santo Tomas, C.P. 11340 Mexico City, Mexico; 4 Banco de Tejidos Radioesterilizados, Instituto Nacional de Investigaciones Nucleares, Carretera México-Toluca S/N La Marquesa, 52750 Ocoyoacac, Mexico; 5 Laboratorio de Nanobiomateriales, Facultad de Estomatología, Universidad Autónoma de San Luis Potosí, Av. Dr. Manuel Nava No. 2, Zona Universitaria, C.P. 78290 San Luis Potosí, Mexico; 6 Laboratorio de Microbiología, Facultad de Ciencias Químicas, Universidad Autónoma de San Luis Potosí, Av. Dr. Manuel Nava No. 6, Zona Universitaria, C.P. 78210 San Luis Potosí, Mexico

**Keywords:** Silver nanoparticles, Radioesterilized pig skin, Mesenchymal stem cells, Anti-biofilm nanocomposites for wound healing

## Abstract

**Background:**

Treatment of severe or chronic skin wounds is an important challenge facing medicine and a significant health care burden. Proper wound healing is often affected by bacterial infection; where biofilm formation is one of the main risks and particularly problematic because it confers protection to microorganisms against antibiotics. One avenue to prevent bacterial colonization of wounds is the use of silver nanoparticles (AgNPs); which have proved to be effective against non-multidrug-resistant and multidrug-resistant bacteria. In addition, the use of mesenchymal stem cells (MSC) is an excellent option to improve wound healing due to their capability for differentiation and release of relevant growth factors. Finally, radiosterilized pig skin (RPS) is a biomatrix successfully used as wound dressing to avoid massive water loss, which represents an excellent carrier to deliver MSC into wound beds. Together, AgNPs, RPS and MSC represent a potential dressing to control massive water loss, prevent bacterial infection and enhance skin regeneration; three essential processes for appropriate wound healing with minimum scaring.

**Results:**

We synthesized stable 10 nm-diameter spherical AgNPs that showed 21- and 16-fold increase in bacteria growth inhibition (in comparison to antibiotics) against clinical strains *Staphylococcus aureus* and *Stenotrophomonas maltophilia,* respectively. RPS samples were impregnated with different AgNPs suspensions to develop RPS-AgNPs nanocomposites with different AgNPs concentrations. Nanocomposites showed inhibition zones, in Kirby–Bauer assay, against both clinical bacteria tested. Nanocomposites also displayed antibiofilm properties against *S. aureus* and *S. maltophilia* from RPS samples impregnated with 250 and 1000 ppm AgNPs suspensions, respectively. MSC were isolated from adipose tissue and seeded on nanocomposites; cells survived on nanocomposites impregnated with up to 250 ppm AgNPs suspensions, showing 35% reduction in cell viability, in comparison to cells on RPS. Cells on nanocomposites proliferated with culture days, although the number of MSC on nanocomposites at 24 h of culture was lower than that on RPS.

**Conclusions:**

AgNPs with better bactericide activity than antibiotics were synthesized. RPS-AgNPs nanocomposites impregnated with 125 and 250 ppm AgNPs suspensions decreased bacterial growth, decreased biofilm formation and were permissive for survival and proliferation of MSC; constituting promising multi-functional dressings for successful treatment of skin wounds.

**Electronic supplementary material:**

The online version of this article (10.1186/s12951-017-0331-0) contains supplementary material, which is available to authorized users.

## Background

Severe acute skin lesions such as those caused by burns, along with chronic wounds, represent a worldwide health care problem [1, 2]. The American Burn Association estimates that about 500,000 patients who suffered burns in the United States in 2013 required specialized medical attention [3]. Second-deep and third degree burns can have devastating lifelong functional and aesthetic sequels if not properly treated [4]; different surgical techniques such as non-viable tissue excision, followed by some type of skin graft constitute the primary treatment option for these lesions. Currently, multi-layered autologous skin grafts are considered the “gold standard” therapy for burn-excised wounds. However, autograft donor sites are quite limited in patients with burns involving more than 50% of the total body surface area, representing one of the main driving-reasons to develop alternative wound dressings [5]. The skin is the largest organ in the human body, spanning approximately 2 m^2^ in an average adult, and has important functions, among which, protection against microorganisms invasion is one of the main ones [6, 7]. Loss of skin integrity exposes subcutaneous tissue to planktonic bacteria colonization, a significant risk for major infection because subcutaneous tissue provides appropriate moisture, temperature and nutrition for rapid bacterial growth and proliferation [8]. Untreated infected wounds promote development of bacterial biofilm, a community of microorganisms attached to a surface and encased within an extracellular polymeric substance that confers microorganisms protection and longevity [9, 10]; biofilm formation is one of the main causes of antibiotic treatment failure [11]. Additionally, the overuse and misuse of antibiotics has significantly contributed to generate multidrug-resistant bacteria strains. Thus, many antibacterial studies have been directed towards the development of new bactericidal compounds [12, 13]. Silver nanoparticles (AgNPs) represent a very good option as topical antibacterial agents to treat locally infected lesions or to prevent wound infections [14–17]. Because of their size, AgNPs can penetrate the bacterial wall, affecting its integrity and consequently, the viability of bacteria. Moreover, AgNPs also generate reactive oxygen species (ROS) which can bind to different proteins, altering bacterial metabolism [18].

Besides preventing and treating bacterial infections, appropriate wound treatment involves other processes such as control of massive loss of water, improvement of neo-vasculature formation, enhancement of dermis and epidermis regeneration and reduction of inflammation [19]. In this sense, it has been shown that adipose-derived mesenchymal stem cells (ADMSC) can differentiate into cell phenotypes that can contribute to restore the skin structure [20]. Moreover, ADMSC secrete Interleukin 10 (IL10), an anti-inflammatory cytokine related to tissue regeneration, and different growth factors correlated to proper wound healing, such as VEGF, EGF and TGF-b [21, 22]. Pig skin has been widely used as a skin dressing for burned patients, because it is biocompatible and decreases massive loss of water and risk of infection [23, 24]. Additionally, pig skin can function as an appropriate cell carrier to transport ADMSC to wound beds and, once in the wound, it can also function as an extracellular matrix-like scaffold to improve skin regeneration [25, 26]. For the safe use of radiosterilized pig skin (RPS), gamma irradiation guarantees that it does not transmit infections, and to the best of our knowledge, there are no reports of rejection when RPS has been used. Gamma-sterilization was first approved in 1963 by British Pharmacopeia and it is currently the most common method for sterilization of tissue allographs [27].

In the present study, we report the synthesis and characterization of AgNPs that were used to impregnate RPS samples, to generate nanocomposites (RPS-AgNPs) that simultaneously displayed antimicrobial properties and were permissive for ADMSC culture. Cellular nanocomposites (RPS-AgNPs-ADMSC) developed in the present study had the potential to simultaneously enhance different processes relevant for appropriate wound healing. They had the potential of functioning as covers to control massive water loss, barriers to prevent bacterial infection and biofilm formation and extracellular-matrix-like structures to improve cell migration and attachment, also providing an appropriate mean for carrying ADMSC into wound beds, which once in there, can secrete molecules relevant for wound healing such as IL10. Thus, RPS-AgNPs-ADMSC nanocomposites constitute integral and promising antimicrobial cellular dressings for treatment of skin wounds.

## Methods

### Synthesis and characterization of silver nanoparticles (AgNPs)

Silver nanoparticles were synthesized following the procedure reported by Pérez-Díaz et al. [28]. Briefly, 100 mL of 10 mM AgNO_3_ solution was mixed with 0.1 g of gallic acid dissolved in 10 mL of deionized water. Then, pH was immediately adjusted to 11 and after reaction, obtained suspensions were dialyzed to purify the AgNPs.

AgNPs were characterized by dynamic light scattering (DLS) to determine their hydrodynamic diameter and zeta potential. Samples were analyzed by triplicate using a Malvern Zetasizer Nano ZS (Malvern Instruments Worcestershire, United Kingdom) operating with He–Ne laser at a wavelength of 633 nm and detection angle of 90°; measurements were performed for 60 s at 25 °C. Vis–NIR absorption spectra were obtained using a CHEMUSB4-VIS–NIR (Ocean optics) spectrophotometer to determine the surface plasmon resonance (SPR) of the AgNPs. Transmission Electron Microscopy (TEM) was used to determinate nanoparticles shape. AgNPs suspensions were diluted with deionized water, 50 μL aliquots were placed on a copper grid for TEM and analyzed using a transmission electron microscope JEOL JEM-1230 (Tokyo, Japan) at an accelerating voltage of 100 kV.

### Bacteria strains

Minimum inhibitory concentrations (MIC) of AgNPs in solution were measured against reference ATCC bacteria strains, *Escherichia coli* (ATCC 25922) and *Enterococcus faecalis* (ATCC 29212), and against clinical bacteria strains *Stenotrophomonas maltophilia* (HCR-392861) and *Staphylococcus aureus* (INR-16-1700). Clinical bacteria strains were isolated from burned patients according to ethic guidelines, maintained in solidified broth (1.5% agar trypticase soy) for 24 h in stagnant condition and identified with a VITEK^®^ system; bacteria susceptibility to antibiotics was tested using the same system. The antibacterial activity of RPS-AgNPS nanocomposites against clinical bacteria strains *S. maltophilia* (HCR-392861) and *S. aureus* (INR-16-1700) was studied by the Kirby–Bauer assay and by slightly modifying the colony biofilm model. Quality of Kirby–Bauer assays performed was corroborated testing reference ATCC bacteria strains (ATCC 25922 and ATCC 25923) against sensidiscs (Becton–Dickinson) impregnated with reference antibiotics according to CLSI 2016 [29].

### Minimum inhibitory concentrations of AgNPs

The microdilution method was used to estimate minimum inhibitory concentrations of AgNPS in solution against clinical bacteria strains *S. maltophilia* (HCR-392861) and *S. aureus* (INR-16-1700), and reference bacteria strains *E. coli* (ATCC 25922) and *E. faecalis* (ATCC 29212). MIC were measured on 96-well microplates according to published protocols [30]. In brief, *S. maltophilia, S. aureus*, *E. coli* and *E. faecalis* were independently incubated in 96-well microplates in a humidified atmosphere for 24 h. Microorganisms were then exposed to serial dilutions of AgNPs solutions from 0.062 to 32 μg/mL, and end points (that is MIC) were determined when no turbidity in the well was observed. The antibacterial activity of the AgNPs was compared to that of commercial antibiotics oxacillin and ceftazidime against *S. aureus* and *E. faecalis*, and *S. maltophilia* and *E. coli,* respectively. Turbidity background from AgNPs solutions was subtracted from final readings. All assays were carried out by triplicate.

### Development and characterization of radiosterilized pig skin-AgNPs (RPS-AgNPs) nanocomposites

Radioesterilized pig skin was kindly supplied by the Banco de Tejidos Radioesterilizados in Mexico (BTR) of the Instituto Nacional de Investigaciones Nucleares (ININ, México). The BTR has a sanitary license for tissue processing since July 7, 1999 and its Quality Management System is certified by ISO 9001:2008 since August 1, 2003 [31]. The BTR has processed and radiosterilized pig skin since 2001, and these tissues have been successfully used as wound dressings in patients from several hospitals in México. In general, tissue processing was as follows: animals were selected at the authorized slaughterhouse, once tissues were in the BTR, they were washed, dried, cut, packed, labeled and subjected to final sterilization using the ININ’s industrial ^60^Co gamma irradiator at 25 kGy. After that, radiosterilized tissues underwent a sterility test as final products and were used in the present experiments [32]. To generate the RPS-AgNPs nanocomposites, RPS circular samples of 0.5 cm in diameter were independently incubated in 10 mL of 125, 250, 500 or 1000 ppm AgNPs suspensions using 40 kHz sonication during 10 min. Then, samples were incubated at room temperature in an orbital shaker at 250 rpm during 24 h. Finally, RPS samples impregnated with AgNPs (RPS-AgNPs nanocomposites) were dried in a type A2 biological safety cabinet for 2 h and named as RPS-AgNPs-125, RPS-AgNPs-250, RPS-AgNPs-500 and RPS-AgNPs-1000, according to the concentration (in ppm) of the AgNPs suspension used during the impregnation process. RPS samples with no AgNPs, simply named as RPS, were used as comparison controls for antibacterial and ADMSC studies.

RPS-AgNPs nanocomposites were studied by electron-dispersive X-ray spectroscopy (EDS) and scanning electron microscopy (SEM) to characterize its elemental composition, morphology and AgNPs surface distribution. Samples were coated with Au thin films and SEM micrographs were acquired in a scanning electron microscope JEOL 7600 at 10 kV in secondary and backscattered electrons modes. Au contributions were removed from acquired EDS spectra and elemental compositions after Au subtraction are presented.

### Evaluation of Ag release from RPS-AgNPs nanocomposites

Silver (AgNPs and Ag ions) release from RPS-AgNPs nanocomposites was evaluated by UV–Vis spectroscopy and atomic absorption spectrometry (AAS). RPS-AgNPs circular samples, 2 cm in diameter, were independently incubated in 2 mL of deionized water at 37 °C and silver release was evaluated every 24 h for 5 days from independent samples incubated for each selected period of time. Experiments were performed by triplicate in two independent assays and RPS samples with no AgNPs were used as controls. At each selected period of time, suspension stocks were collected and absorbance was measured at 420 nm (expected wavelength for the characteristic SPR of AgNPs) in a synergy HTX spectrophotometer. Increasing concentration (from 5 to 50 ppm) AgNPs reference suspensions were also measured to express experimental data as AgNPs concentration in ppm. 1 mL aliquots of experimental suspension stocks were diluted with deionized water to a final volume of 10 mL. Then, silver concentration was determined using an air-acetylene flame atomic absorption spectrometer (Pinaacle 500, Perkin Elmer). Standard reference AgNPs suspensions were also measured by AAS and experimental data are reported as AgNPs concentration (ppm) in the experimental stock solutions.

### Antibacterial properties of nanocomposites

The Kirby–Bauer method was used to study bacterial inhibition zones due to, mainly, diffusion of silver nanoparticles on solid medium from nanocomposites. To characterize bacterial inhibition zones of nanocomposites, clinical bacteria strains, *S. maltophilia* (HCR-392861) and *S. aureus* (INR-16-1700), were uniformly inoculated on petri dishes with Mueller–Hinton agar to a concentration of 1:1000, starting from a 0.5 McFarland bacterial solutions Then, inoculum was allowed to dry for 5–20 s. Subsequently, RPS (as negative controls; no antibacterial effect expected) and RPS-AgNPs nanocomposites samples were independently placed on inoculated petri dishes and incubated for 24 h at 37 °C. After incubation, nanocomposites inhibition zones were measured and compared with those of RPS.

The capability of RPS-AgNPs nanocomposites to inhibit biofilm formation was studied by slightly modifying the colony biofilm model [33]. Briefly, RPS-AgNPs samples of 0.5 cm in diameter were independently placed on culture wells with agar trypticase soy medium and inoculated with 7 µL of diluted 1:1000 stationary phase planktonic culture suspension (microorganism suspensions at optical density of 0.08 at 600 nm); final concentration after dilution was approximately 1.5 × 10^5^ microorganisms per milliliter. RPS samples of 0.5 cm in diameter were used as antibacterial negative controls (no antibacterial effect expected) and all experiments were performed by triplicate independently for *S. maltophilia* and *S. aureus*. After 24 h of incubation, samples were removed and rinsed in 180 µL of saline solution. Biofilms formed on RPS an RPS-AgNPs samples were disaggregated using a sequence of treatments, which included vortexing (Vortex Genie 2; Scientific Products) and sonication (model 2510 sonicating water bath; Branson), alternating 120 s of sonication at 42 kHz with 30 s of vortexing, according to published protocols [34]. Suspensions of bacteria obtained from biofilms disaggregation were serially diluted in 0.85% saline solution, in 10 consecutive dilutions from 1 × 10^−3^ to 1 × 10^−10^ v/v. Dilutions were named consecutively as 3, 4, 5, 6, 7, 8, 9 and 10, plated by triplicate on agar trypticase soy plates and incubated for 24 h at 37 °C. Finally, colony-forming units (CFU) from dilution 8 (individual CFU clearly observed for all samples) were counted.

### Isolation of adipose-derived mesenchymal stem cells

Isolation of adipose-derived mesenchymal stem cells **(**ADMSC) was performed as previously reported [35]. The consent and experimental protocols in this study were reviewed and approved by the ethics committee of the Instituto Nacional de Rehabilitación Luis Guillermo Ibarra Ibarra (México). In brief, subcutaneous adipose tissue was obtained from aesthetic surgeries of patients undergoing elective liposuction; all samples were recollected previous signature of informed consent. Liposuctions were carried out using a needle with an internal diameter of 4 mm. Adipose tissue was digested with 0.1% type I collagenase (Worthington Biochemical) in DMEM (Dulbecco’s Modified Eagle Medium; GIBCO) during 45 min at 37 °C with shaking at 200 rpm. Cells suspension was passed through a 70 µm strainer and centrifuged at 1200 rpm for 5 min. Cells were collected, resuspended and seeded in tissue culture plates at 50,000 cells per cm^2^. After 24 h of culture, medium was changed and adherent ADMSC were cultured to confluence as primary culture. Cells were maintained in DMEM supplemented with 10% FBS (fetal bovine serum; GIBCO) and 1% penicillin/streptomycin (GIBCO).

### Generation and evaluation of cellular constructs (ADMSC on nanocomposites)

To study the suitability of RPS-AgNPs nanocomposites for cellular culture, ADMSC at passage one were collected and seeded onto RPS and RPS-AgNPs nanocomposites samples of 0.5 cm in diameter. Each sample was seeded with ADMSC at a concentration of 30,000 cells/cm^2^ to form what was called a construct. Finally, constructs were cultured in DMEM complemented with 10% FBS and 1% penicillin/streptomycin at 37 °C and 5% CO_2_ for up to 8 days; culture medium was changed every 2 days.

We used the LIVE/DEAD^®^ Viability/Cytotoxicity for mammalian cells Molecular Probes^®^ kit to determine cell viability on the different RPS-AgNPs nanocomposites. Cell viability after 24 h of culture on nanocomposites was determined following technical specifications established by the kit manufacturer. 1 μM calcein AM and 2 μM EthD-1 were diluted in Hank’s medium and this solution was used to incubate the ADMSC-RPS-AgNPs constructs for 45 min at 37 °C. Constructs were washed with PBS and photographed using a confocal microscope LSM 780 and ZEN 2010 Carl Zeiss. Calcein/EtDh-1 (alive/dead) positive cells and total number of cells were counted using the Image J software^®^.

To study ADMSC proliferation on nanocomposites, ADMSC were seeded at a density of 30,000 cells/cm^2^ on RPS and RPS-AgNPs nanocomposite samples and cultured at 37 °C and 5% CO_2_; seeding point was considered as culture day 0. Cells were detached and counted every day, from independently but simultaneously seeded samples, until culture day 5. Cells were detached from RPS and RPS-AgNPs samples using 0.25% trypsin solution during 10 min at 37 °C. Detached cells were counted with a hemocytometer chamber to indirectly study cell proliferation on RPS and nanocomposite samples.

To assess the potential cytotoxic effect of silver (AgNPs and Ag ions) release, from RPS-AgNPs nanocomposites, on ADMSC over time, ADMSC were seeded in well culture plates at 5260 cell/cm^2^ and cultured for 24 h in DMEM complemented with 10% FBS and 1% penicillin/streptomycin at 37 °C and 5% CO_2_. Simultaneously, RPS and RPS-AgNPs nanocomposite circular samples, 2 cm in diameter, were independently incubated with 2 mL of DMEM complemented with 10% FBS and 1% penicillin/streptomycin. After the first 24 h of cell culture (time 0), culture medium was removed and replaced with supernatants collected from incubated RPS or RPS-AgNPs samples every 24 h; fresh aliquots of complemented DMEM were added to the RPS and RPS-AgNPs samples in incubation. This procedure was repeated every day for up to 4 days using independent ADMSC samples for each selected period of time (1, 2, 3 and 4 days). Controls corresponded to cells samples cultured with fresh DMEM complemented with 10% FBS and 1% penicillin/streptomycin. Cytotoxicity of RPS and nanocomposites supernatants was evaluated at each selected period of time using the colorimetric MTT (3-(4,5-dimethylthiazol-2-yl)-2,5-diphenyl tetrazolium bromide) assay. For this purpose, after corresponding exposure to supernatants, cells were rinsed with PBS and incubated with MTT:DMEM solution (1:10) for 3 h. Then, cells metabolized formazan crystals were solubilized in 2-propanol:dimethyl sulfoxide (1:1) and absorbance was measured at 570 nm (Synergy HTX spectrophotometer). Experiments were independently performed by triplicate for each selected period of time and RPS or RPS nanocomposite sample evaluated.

### Statistical analysis

All experiments were repeated at least three times. RPS and RPS-AgNPs nanocomposites samples were tested in parallel with the same batch of cells or bacteria. Results are expressed as the mean ± standard error. ANOVA followed by Tukey’s multiple comparison tests were used to compare more than two populations. A p value ≤ 0.05 was considered statistically significant.

## Results

### Characterization of AgNPs

**Fig. 1:**
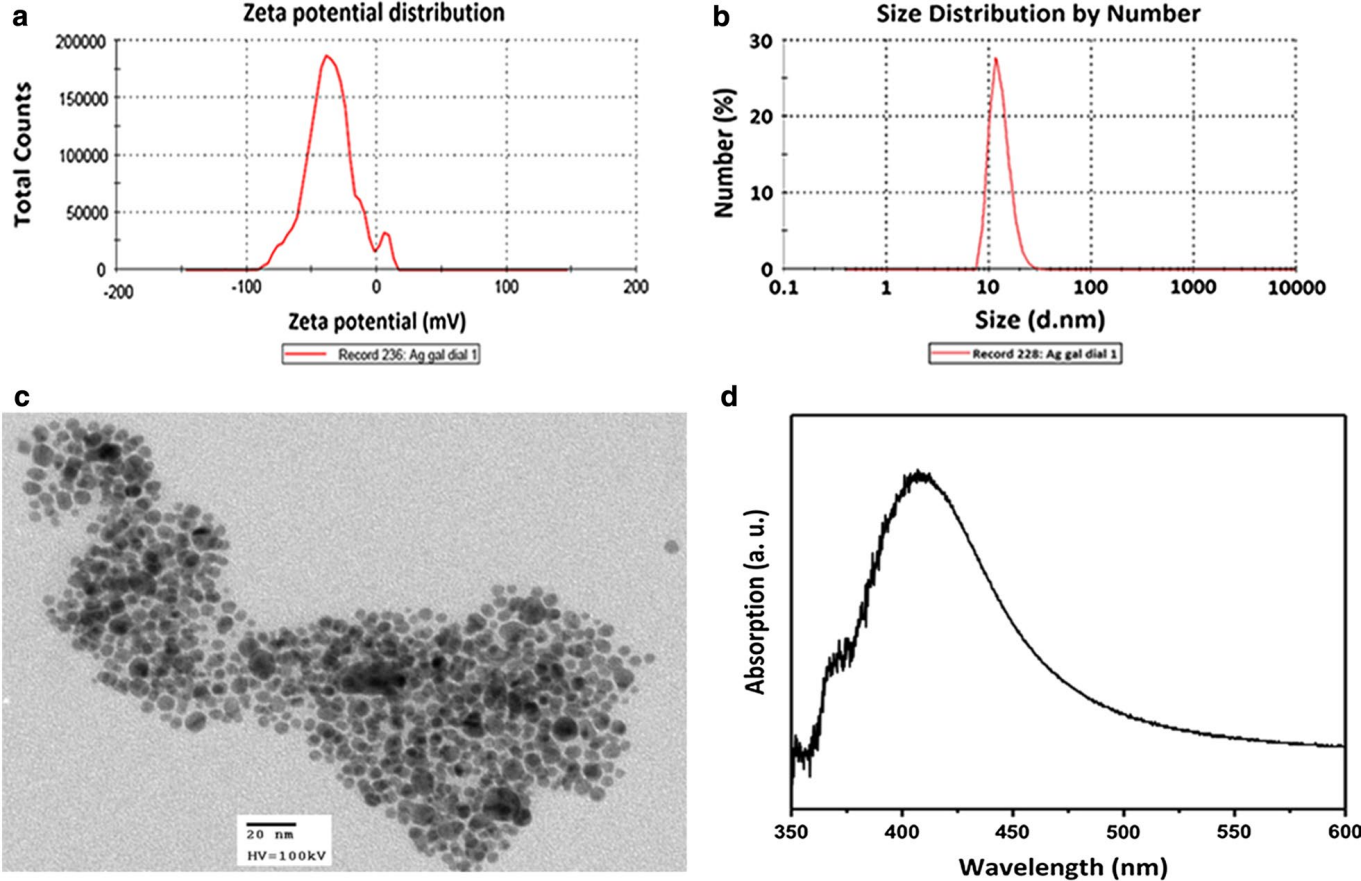
Physical characterization of silver nanoparticles (AgNPs). **a** Zeta potential, **b** hydrodynamic diameter (dynamic light scattering), **c** general shape (transmission electron micrograph) and **d** surface plasmon resonance (SPR) values for synthesized AgNPs

In the present synthesis of AgNPs, gallic acid was used as reducing and stabilizing agent to improve the synthesis of stable spherical-like AgNPs with small average diameter (≈ 10 nm) and narrow size distribution (Fig. 1). AgNPs were analyzed by TEM, and results confirmed that synthesized silver nanoparticles were well dispersed and presented pseudo-spherical shapes (Fig. 1c). DLS showed AgNPs with average hydrodynamic diameter equal to 13.03 ± 1.65 nm and narrow size distribution of 9–20 nm (Fig. 1b). A surface plasmon resonance (SPR) was observed at 420 nm, corresponding to the characteristic SPR of silver nanoparticles (Fig. 1d). Zeta potential values ≈ − 38 ± 8 mV were obtained for AgNPs, confirming the synthesis of stable nanoparticles (Fig. 1a).

### Antimicrobial activity of AgNPs in solution

Clinical bacteria strains, *S. aureus* and *S. maltophilia*, (Gram-positive and Gram-negative; respectively), were isolated from infected burned patients of the Instituto Nacional de Rehabilitación and Hospital Dr. Ignacio Morones Prieto (Mexico) and coded as follows: INR-16-1700 for *S. aureus* and HCR-392861 for *S. maltophilia*. Antibiograms performed using the VITEK^®^ system showed both bacteria strains as multi-resistant microorganisms.

The microdilution method was used for estimation of minimum inhibitory concentrations (MIC), showing that AgNPs in solution had antimicrobial activity (from low concentrations) against the two clinical multi-resistant bacteria strains studied in the present work; INR-16-1700 and HCR-392861 (Table 1). MIC results also showed that AgNPs exhibited a better bactericide effect than antibiotics, oxacillin and ceftazimide, against INR-16-1700 (Gram positive) and HCR-392861 (Gram negative), respectively. Bactericide effect of AgNPs in solution showed a 21-fold and a 16-fold increase, against INR-16-1700 and HCR-392861, respectively, in comparison to antibiotics tested in the present study. MIC of AgNPs in solution against Gram-negative *S. maltophilia* (HCR-392861) was smaller than MIC determined for Gram-positive *S. aureus* (INR-16-1700); Table 1.

**Table 1:** Minimum inhibitory concentrations

Bacteria	Antibiotic or AgNPs	MIC ± SD (µg/mL)
*Stenotrophomonas maltophilia* HCR-392861^G−^	AgNPs	4.66 ± 1.15
	Ceftazidime	74.66 ± 18.47
*Escherichia coli* ATCC 25922^G−^	AgNPs	4.00 ± 0.0
	Ceftazidime	0.25 ± 0.0
*Staphylococcus aureus* INR-16-1700^G+^	AgNPs	6.00 ± 2.00
	Oxacillin	128.00 ± 0.0
*Enterococcus faecalis* ATCC 29212^G+^	AgNPs	7.30 ± 1.15
	Oxacillin	9.30 ± 2.30

Table shows minimum inhibitory concentrations of silver nanoparticles in solution and of antibiotics against clinical bacteria strains, HCR-392861 and INR-16-1700, and against reference bacteria strains ATCC 25922 and ATCC 29212

*AgNPs* silver nanoparticles, *MIC* minimum inhibitory concentration, *SD* standard deviation, *G*^*+*^ Gram-positive bacteria and *G*^*−*^ Gram-negative bacteria

### RPS-AgNPs nanocomposites characterization

Once independent RPS samples were impregnated with different AgNPs concentrations to obtain the set of RPS-AgNPs nanocomposites of study, they were characterized by SEM and EDS, and their silver release upon incubation in water was characterized by UV–Vis spectroscopy and AAS. Electron-dispersive X-ray spectroscopy studies were performed to measure the amount of Ag in the different RPS-AgNPs nanocomposites. Results are summarized in Table 2 showing the average elemental composition (atomic percentage, at.%) of the different nanocomposites. Atomic percentage of Ag in nanocomposites increased as higher AgNPs concentration solutions were used for impregnation of RPS samples. Noteworthy is the fact that RPS contained N and S, elements that were not present in RPS-AgNPs-250, RPS-AgNPs-500 or RPS-AgNPs-1000. Sulfur and nitrogen were present in RPS-AgNPs-125; however, atomic percentage of S in this sample was significantly smaller than that in RPS. It is also important to mention that RPS-AgNPs-500 showed a particularly less-uniform surface distribution of AgNPs. Consequently, standard deviation (SD) of Ag at.% for this nanocomposite was significantly higher (1.16 ± 1.083 at.%) due to the presence of low and high Ag at.% areas (Ag ≈ 0.26 and 2.47 at.%; respectively) coexisting within the samples.

**Table 2:** Elemental composition of RPS and RPS-AgNPs nanocomposites as measured from electron-dispersive X-ray spectroscopy (EDS)

Sample	C (at.%)	O (at.%)	N (at.%)	Ag (at.%)	S (at.%)
RPS	54.44 ± 0.39	25.15 ± 0.18	19.98 ± 0.32	0.0	0.37 ± 0.02
RPS-AgNPs-125	65.09 ± 2.44	26.25 ± 2.40	8.03 ± 6.03	0.59 ± 0.28	0.32 ± 0.24
RPS-AgNPs-250	64.64 ± 1.07	29.84 ± 1.68	0.00	0.91 ± 0.19	0.00
RPS-AgNPs-500	72.17 ± 3.46	26.74 ± 3.46	0.00	1.16 ± 1.083	0.00
RPS-AgNPs-1000	64.90 ± 2.65	29.55 ± 2.65	0.00	5.65 ± 3.84	0.00

Data are presented as mean ± standard deviation after Au subtraction from EDS spectra

*at.%* atomic percentage, *C* carbon, *O* oxygen, *N* nitrogen, *Ag* silver, *S* sulfur

**Fig. 2:**
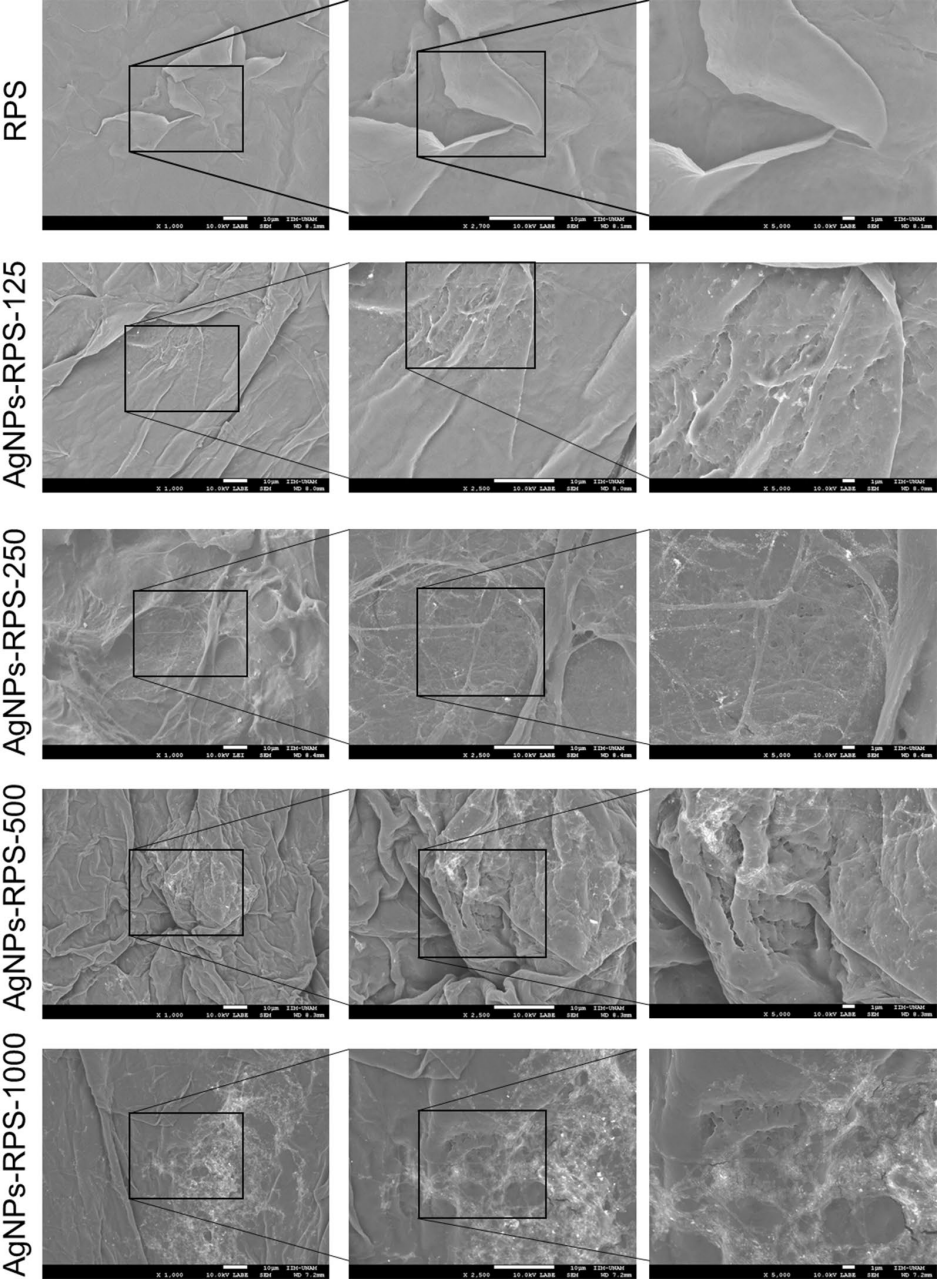
Characterization of RPS and RPS-AgNPs nanocomposites by scanning electron microscopy (SEM). Figure shows representative SEM micrographs (backscattered electrons) of RPS and RPS-AgNPs nanocomposites samples with different AgNPs concentration

Representative micrographs of RPS and RPS-AgNPs nanocomposite samples are shown in Fig. 2. All nanocomposites presented a rougher surface than RPS; however, roughness was similar for all nanocomposites (RPS-AgNPs samples) independently of AgNPs concentration impregnated on RPS. That is, the same general roughness increment in comparison with RPS was observed for all RPS-AgNPs nanocomposites, independently of its silver atomic percentage (at.%). Surface-distribution of impregnated AgNPs was not completely homogeneous and silver nanoparticles seemed to concentrate on the rougher or more uneven surface areas. Nevertheless, these rougher surface areas with higher AgNPs concentration were homogeneously distributed throughout the surface of nanocomposites. Within the areas of high silver concentration, AgNPs were well dispersed with no apparent agglomeration effects occurring upon impregnation on RPS.

**Fig. 3:**
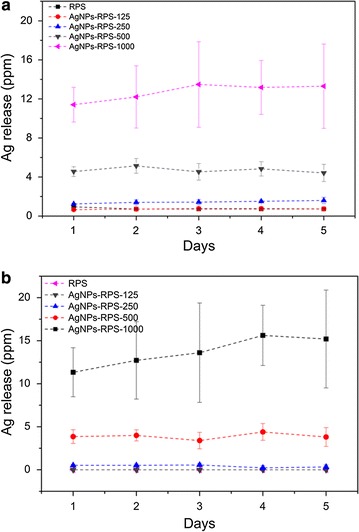
Nanocomposites silver release characterization. Figure shows silver release over time from AgNPs-RPS nanocomposites incubated in deionized water as determined by **a** UV–Vis spectroscopy (420 nm) and **b** atomic absorption spectrometry

Silver release from RPS-AgNPs nanocomposites (3.14 cm^2^ nanocomposite samples incubated in 2 mL of water) is shown in Fig. 3a, b). Silver release from RPS-AgNPs-125 was below the concentration range of detection for UV–Vis spectroscopy and AAS. Silver release corresponded to ≈ 0.7 (1.4), 4.1 (4.6) and 13.5 (12.8) ppm as measured from AAS (UV–Vis spectroscopy), respectively for AgNPs-RPS-250, AgNPs-RPS-500 and AgNPs-RPS-1000. The higher silver release occurred during the first 24 h of incubation for all nanocomposites and it seemed that after that period of time non-significant further silver release occurred. Average silver release from AgNPs-RPS-1000 seemed to be constant over the first 4 (3) incubation days as determined from AAS (UV–Vis spectroscopy) measurements; however, differences were not statistically significant.

### Nanocomposites antibacterial activity

**Fig. 4:**
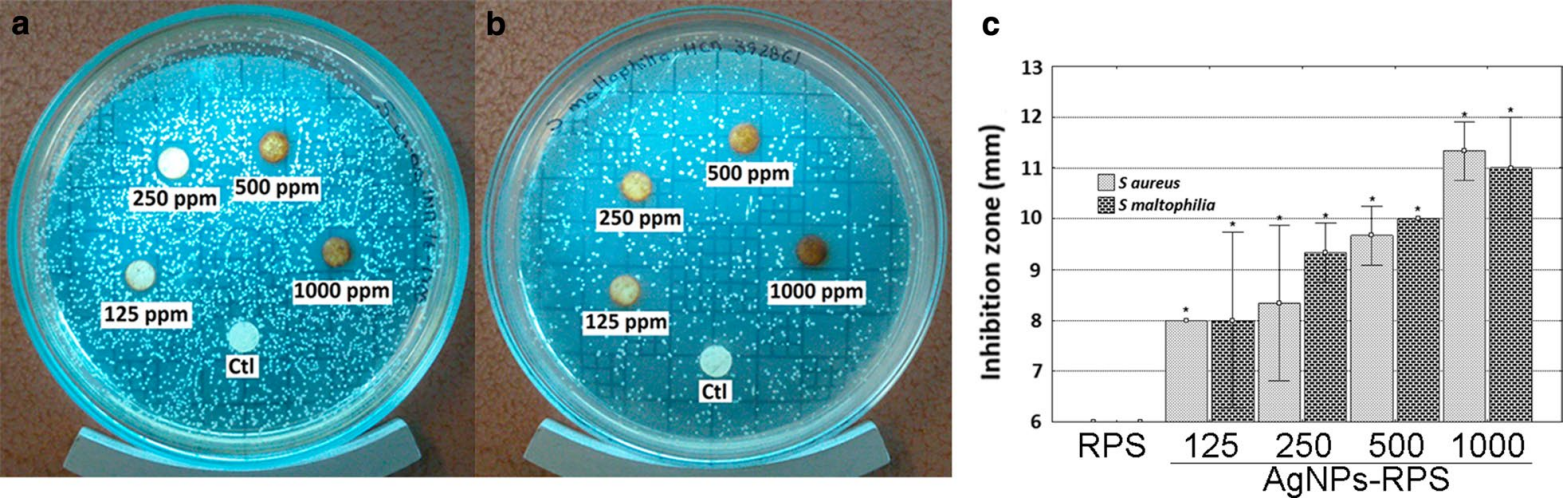
Inhibition halos of RPS-AgNPs nanocomposites by Kirby–Bauer assay. For both bacterial strains tested **a** *Staphylococcus aureus* (INR-16-1700) and **b** *Stenotrophomonas maltophilia* (HCR-392861), inhibition halos were dependent on AgNPs concentration of nanocomposites. Inhibition halos increased with increasing AgNPs concentration. **c** All nanocomposites (RPS-AgNPs-125, RPS-AgNPs-250, RPS-AgNPs-500 and RPS-AgNPs-1000) showed significantly higher inhibition halos than RPS; *p < 0.05 ANOVA, Tukey pos hoc test

Kirby–Bauer assays were performed to evaluate the antibacterial effect of nanocomposites on bacteria growth. Cultures of clinical bacteria strains (*S. aureus*; INR-16-1700, and *S. maltophilia*; HCR-3928) isolated from burned patients were treated with RPS and RPS-AgNPs samples in the Kirby–Bauer assay (Fig. 4) to study the antibacterial activity of nanocomposites against bacteria strains related to common infections in burned patients. Nanocomposites showed inhibition halos ≈ 8 to 11 mm in diameter depending on Ag concentration of nanocomposites. Inhibition halos for the same nanocomposite were of similar diameter against both clinical bacteria strains *S. maltophilia* and *S. aureus* (HCR-392861 and INR-16-1700, respectively); Table 3. All nanocomposites (RPS-AgNPs-100, RPS-AgNPs-250, RPS-AgNPs-500 and RPS-AgNPs-1000) inhibited the growth of *S. aureus* and *S. maltophilia*, while RPS did not inhibit the growth of any of these two bacteria strains. Differences between inhibition halos showed by nanocomposites and RPS were statistically significant; p ≤ 0.05. RPS-AgNPs-1000 displayed the largest inhibition halos (Fig. 4 and Table 3).

**Table 3:** Inhibition halos of RPS and RPS-AgNPs nanocomposites

Sample	Bacteria	
	*Staphylococus aureus* (INR-16-1700)	*Stenotrophomonas maltophilia* (HCR-392861)
	Inhibition halos (mm) ± SD	
RPS	0	0
RPS-AgNPs-125	8 ± 0	8 ± 1.73
RPS-AgNPs-250	8.33 ± 1.53	9 ± 0.58
RPS-AgNPs-500	9.67 ± 0.58	10 ± 0
RPS-AgNPs-1000	11.33 ± 0.58	11 ± 1

*RPS* radiosterilized pig skin, *RPS-AgNPS* radiosterilized pig skin-Ag nanoparticles, *SD* standard deviation

### Anti-biofilm activity of nanocomposites

**Fig. 5:**
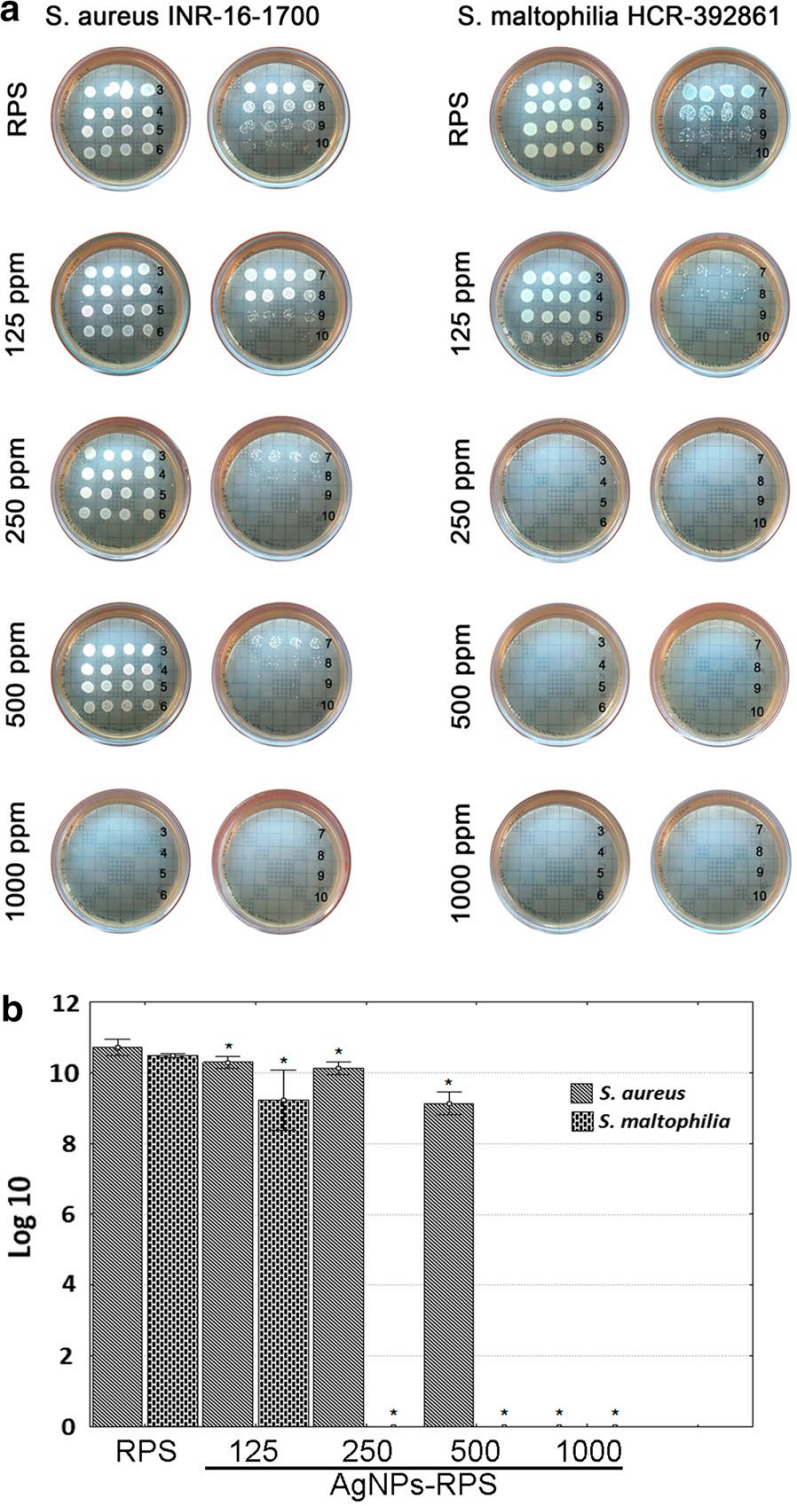
Anti-biofilm activity of RPS-AgNPs nanocomposites. **a** Left-side set of photographs shows nanocomposites inhibition of biofilm formation against *Staphylococus aureus* (INR-16-1700), while; right-side set of photographs shows the inhibition effect of nanocomposites against *Stenotrophomona maltophila* (HCR-392861). Numbers on each photograph represent inoculum (biofilm disaggregation from nanocomposites) serial dilutions in saline solution, from 3 representing 1 × 10^−3^ v/v dilution to 10 representing 1 × 10^−10^ v/v dilution. **b** Graph shows quantitative analysis of biofilm inhibition against *S. aureus* (INR-16-1700) and *S. maltophilia* (INR-16-1700), presenting the Log 10 of the number of colony forming units (CFU) counted from dilution 8 vs nanocomposite from where biofilm was disaggregated); *p < 0.05 ANOVA, Tukey pos hoc test

The capability of RPS-AgNPs nanocomposites to inhibit biofilm formation was also evaluated, showing that nanocomposites presented different anti-biofilm activity, against the clinical bacteria strains tested, depending on bacteria strain and AgNPs concentration in nanocomposites. After 24 h of incubation with bacteria inoculum in liquid media, RPS and nanocomposites samples were rinsed, and bacteria attached to them (initial stage of biofilm formation) were disaggregated, diluted at different concentrations, inoculated on agar plates and culture for 24 h. Anti-biofilm activity of nanocomposites was then indirectly evaluated by the number of colony forming units (CFU) observed (Fig. 4). Against *S. aureus,* INR-16-1700, complete eradication of bacteria was observed for RPS-AgNPs-1000 nanocomposite. *S. maltophilia,* HCR-392861, showed higher susceptibility to nanocomposites and complete bacteria eradication was observed from RPS-AgNPs-250 (Fig. 5).

### Viability and proliferation of ADMSC cultured on nanocomposites

**Fig. 6:**
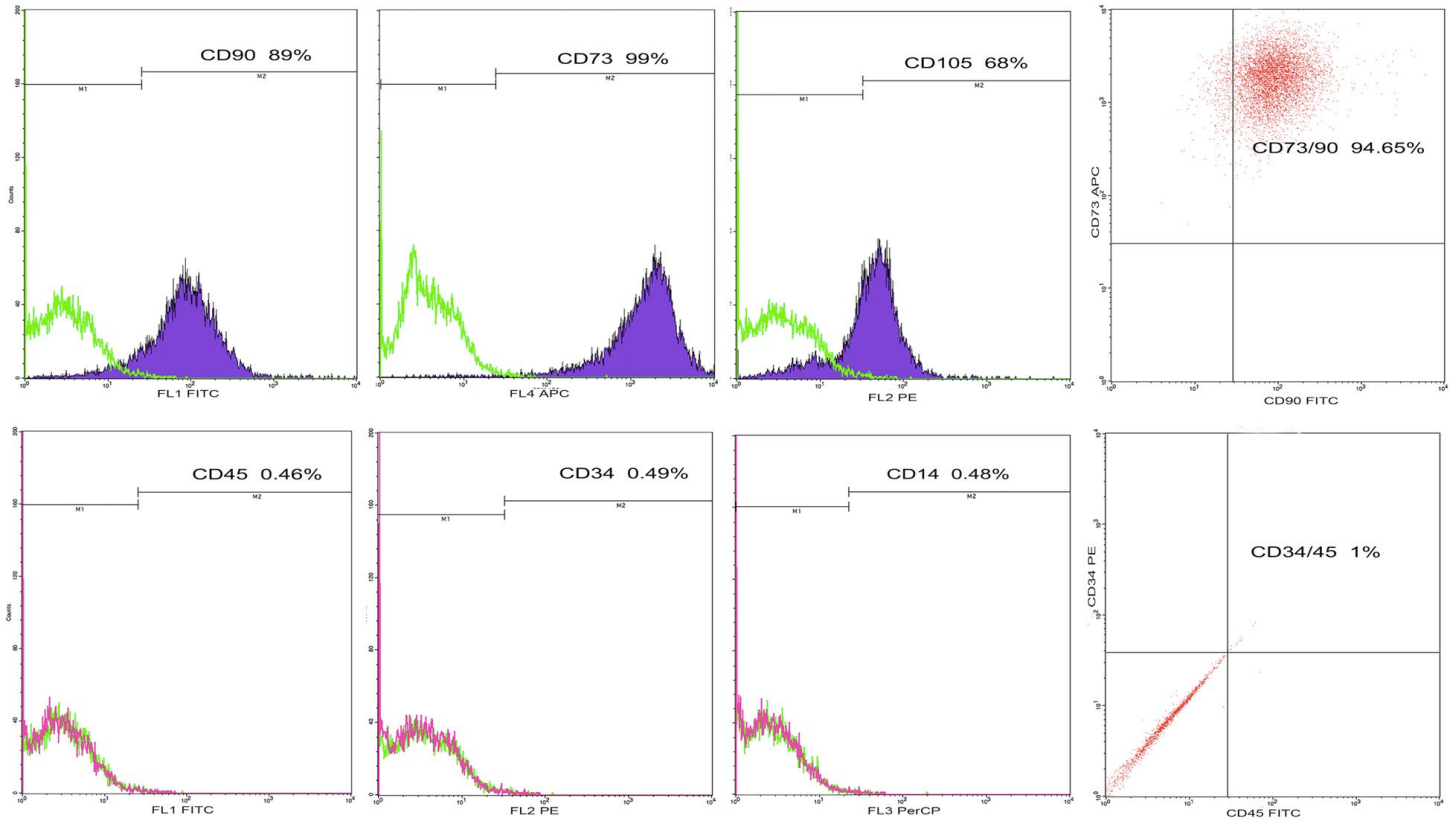
Immunophenotype of human adipose-derived mesenchymal stem cells by flow citometry. Data shown correspond to percentage of human adipose-derived mesenchymal stem cells (ADMSC) labeled with primary monoclonal antibodies conjugated to a fluorochrome. Upper panel graphs show that ADMSC were positive for the expression of mesenchymal stem cells markers, CD90-FITC, CD73-APC, CD105-PE; 94.65% of cells were positive for co-expression of CD73-APC/CD90-FITC. Lower panel graphs show that ADMSC were negative for expression of hematopoietic stem cell markers, CD45FITC, CD34PE and CD14PerCP; only less than 1% of cells were positive for CD45FITC/CD34PE co-expression

It was very important to determine the appropriate AgNPs concentration in RPS-AgNPs nanocomposites that allows culture of MSC. For this purpose, in the present study, ADMSC [35] which positively expressed characteristic cell markers of mesenchymal stem cells (Fig. 6) were used to evaluate the cell culture properties of the nanocomposites.

**Fig. 7:**
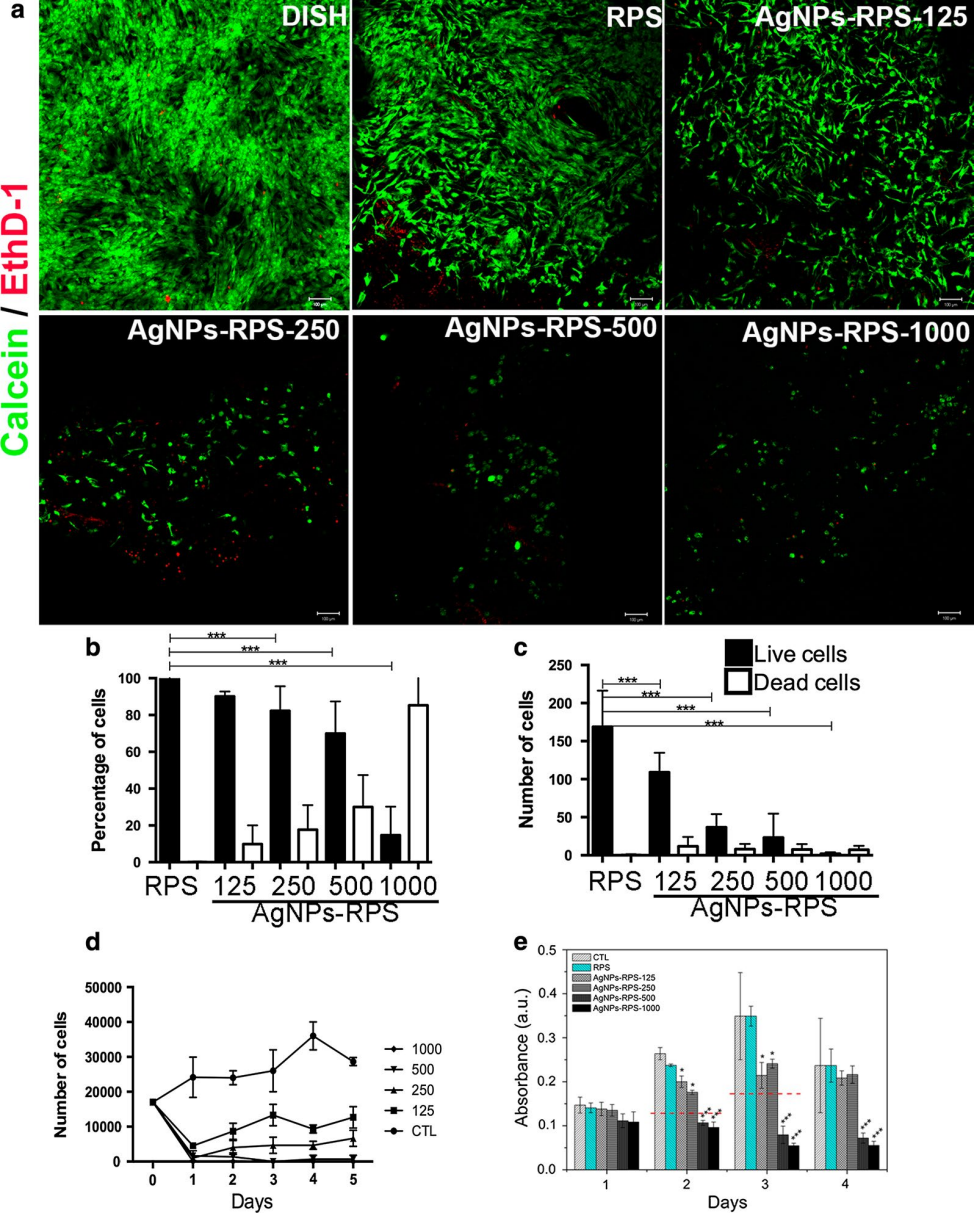
Viability of adipose-derived mesenchymal stem cells cultured on nanocomposites. **a** representative micrographs of cell viability assays at 24 h of culture; live cells (calcein positive cells) are marked in green and dead cells (EthD-1 positive cells) are marked in red. CTL (control) corresponds to cells cultured on culture multiwell plates. Graph **b** shows cell viability percentage for each experimental condition. Graph **c** shows the total number of cells (live and dead) present on CTL, RPS and RPS-AgNPs nanocomposites after 24 h of culture. Graph **d** shows the number of viable cells detached from CTL, RPS and RPS-AgNPs nanocomposites at different culture times (cell proliferation curves), for up to 5 days of culture. Graph **e** shows the amount of metabolically active cells (as indirectly evaluated from absorbance measurements by MTT assay) over time, after cells exposure to RPS or AgNPs-RPS supernatants or CTL (cells exposed to fresh DMEM complemented with 10% FBS and 1% penicillin/streptomycin): For all graphs, ***p < 0.0001vs CTL; ANOVA, Tukey post hoc test

The calcein–ethidium homodimer assay was used to evaluate cell viability upon culture on nanocomposites; Fig. 7. Viability assays performed for cell monolayers on culture multiwell plates (CTL) showed that more than 95% of isolated ADMSC were viable (Fig. 7). At 24 h of culture, ADMSC cultured on RPS exhibited viabilities higher than 95%; while ADMSC on RPS-AgNPs nanocomposites showed viability percentages ≈ 84, 74, 60 and 6%, respectively for RPS-AgNPs-100, RPS-AgNPs-250, RPS-AgNPs-500 and RPS-AgNPs-1000 (Fig. 7). Although the number of viable cells (calcein positive cells) after 24 h of culture on RPS was larger than that on the nanocomposites, it was a smaller than the corresponding number of viable cells on culture-well dishes (Fig. 7b–d). Number of viable cells on RPS-AgNPs-125 decreased 35% in comparison to that on RPS (Fig. 7a, c). RPS-AgNPs-250, RPS-AgNPs-500 and RPS-AgNPs-1000 nanocomposites showed an important decrement of the total number of cells at 24 h of culture, in comparison to RPS (Fig. 7a, c).

It was observed that increasing concentrations of AgNPs in nanocomposites decreased the number of cells present on them at 24 h of culture, in comparison to cells cultured on CTL or RPS; Fig. 7a–c). Thus, cell proliferation over time of ADMSC cultured on nanocomposites was studied (Fig. 7d). Number of cells on RPS increased with culture days (cell proliferation) since culture day 1. On the other hand, cells cultured on nanocomposites showed a reduction in number at culture day 1 in comparison to CTL or RPS; corroborating observations from viability assays (Fig. 7a, b). However, the number of cells on RPS-AgNPs-125 and RPS-AgNPs-250 increased with culture days; indicating that cells remaining on nanocomposites after seeding proliferated with culture time. RPS-AgNPs-500 and RPS-AgNPs-1000 prevented cell proliferation; number of cells remaining on these two nanocomposites after seeding did not significantly increase with culture days. Number of cells on RPS-AgNPs-500 exhibited a small but not significant increment with culture time and, number of cells on RPS-AgNPs-1000 continued minimum and constant for up to 5 days of culture.

The potential cytotoxic effect of silver release, from RPS-AgNPs nanocomposites, on ADMSC was studied over time by MTT assays. Viability (amount of metabolically active cells) of cells exposed to supernatants (silver release) from RPS-AgNPs nanocomposites was not significantly different from CTL or cells exposed to RPS supernatants during the first 24 h of exposure. By day 2 and 3 of exposure, the amount of metabolically active cells increased for cells exposed to supernatants from AGNPs-RPS-125 and AgNPS-RPS-250, in comparison to corresponding samples evaluated at 24 h of exposure to supernatants; however, the amount of metabolically active cells was significantly smaller than that on CTL or cell exposed to RPS supernatants at days 2 and 3 of exposure. These results were in agreement with proliferation assays of ADMSC seeded and cultured on AgNPs-RPS nanocomposites (Fig. 7d). Percentage of metabolically active cells after 2 days of exposure to AgNPs-RPS-125 and AgNPs-RPS-250 supernatants was ≈ 76.9 and 67.7%, respectively, in comparison to CTL (100%), and it was 61.4 and 64.8%, respectively, in comparison to CTL after 3 days of exposure to supernatants. In the case of cells exposed to AgNPs-RPS-500 and AgNPs-RPS-1000 supernatants, the amount of metabolically active cells decreased over exposure time, and it was smaller than 50% in comparison to the amount of metabolically active cells in CTL by days 2 and 3 of exposure. By day 4 of exposure, the amount of metabolically active cells did not further increase in comparison to exposure day 3, in the case of AgNPs-RPS-125 and AgNPS-RPS-250 supernatants; nevertheless, a decrease in the amount of metabolically active cells in CTL was also observed by day 4.

## Discussion

For the synthesis of AgNPs, gallic acid was used as both reducing and stabilizing agent to obtain stable and spherical AgNPs using a fast, environmentally friendly and one-step process. In this green process, oxidation reaction of phenol groups in gallic acid is responsible for silver ions reduction (Additional file 1: Figure S1). Then, produced quinoid compounds with a ketoenol-system are adsorbed on the surface of silver nanoparticles accounting for its stabilization [36, 37]. Additionally, gallic acid molecules on the surface of nanoparticles form hydrogen bonds between neighboring molecules further stabilizing the AgNPs [38]. Reduction reaction was carried out at pH = 11, where phenol groups are expected to be ionized, consequently, reduction reaction occurs very fast, so that spherical nanoparticles are obtained. AgNPs synthesized in the present study with the aforementioned method presented a pseudo-spherical shape with an average diameter ≈ 13 nm, which was corroborated by the defined UV–Vis absorption band at 420 nm assigned to the characteristic SPR of Ag particles with dimensions around 10 nm. AgNPs average diameter and spherical morphology was also corroborated by DLS measurements and TEM. Finally, zeta potential of AgNPs (− 38 mV) indicated that they were stable.

It has been shown by previous studies that particle size and size distribution are two of the most important characteristic of AgNPs and greatly determined their antibacterial activity [39, 40]. It has been reported that AgNPs smaller than 3 nm can be highly cytotoxic even for eukaryote cells or large organisms; while AgNPs between 10 and 20 nm are expected to be cytotoxic for prokaryote cells but not highly cytotoxic for eukaryote cells, representing good nanomaterials to be used as antibacterial compounds for treatments intended to be applied on patients [39, 40]. On the other hand, synthesis of stable silver nanoparticles is a relevant factor because it will prevent their aggregation and the consequently loos of its antibacterial properties; which are highly related to their nano-dimensions. Based on this, physical properties (stability and average diameter ≈ 13 nm) of the present AgNPs made them nanoparticles with potential for developing antibacterial nanocomposites intended to be used as wound dressings. Nanocomposites were developed by impregnating the synthesized AgNPs on RPS samples. pH value of AgNPs in solution after dialysis was around 7.0, which is very close to physiological pH. Thus, treatment of RPS with AgNPs solutions was not expected to induce significant cytotoxic effects due to acid or basic pH ambient during eukaryote cells culture.

Minimum inhibitory concentrations of AgNPs against clinical multi-drug resistant bacteria strains (*S. aureus*, INR-16-1700, and *S. maltophilia*, HCR-392861) were significantly lower than those required for bacteria growth inhibition when using reference antibiotics, ceftamizine or oxacillin, respectively for Gram-negative *S. maltophilia* (HCR-392861) or Gram-positive *S. aureus*, (INR-16-1700); Table 1. In the case of ATCC bacteria strains, *E. coli* (ATCC 25922) and *E. faecalis* (ATCC 29212), MIC of AgNPs were similar to those obtained for antibiotics. However, it is important to mention that, MIC exhibited by the present AgNPs were in the lower range of MIC values previously reported for AgNPs of similar size against ATCC *E. coli* and *S. aureus* strains [41, 42]. MIC values determined for clinical and ATCC bacteria strains in the present report evidenced the potential of the AgNPs synthesized in the present study to prevent/combat multidrug-resistant bacterial infections; which, along with biofilm formation, represent one of the main causes of chronic infection and wound healing impairment. AgNPs MIC measured for Gram-negative *S. maltophilia* (HCR-392861) were smaller than those determined for Gram-positive *S. aureus* (INR-16-1700), probably due to the dense peptidoglycan structure that forms part of the cell wall of Gram-positive bacteria and functions as a protective barrier. Nevertheless, AgNPs MIC against *S. maltophilia* and *S. aureus* were significantly smaller (16- and 20-fold times smaller, respectively) than those measured for reference antibiotics against the same bacteria strains; Table 1.

Successful formation of RPS-AgNPs nanocomposites was corroborated from SEM micrographs that showed a good distribution of well-dispersed AgNPs over the surface of RPS samples (Fig. 2). AgNPs distribution in nanocomposites was quite similar to nanoparticles distribution in commercially available antibacterial dressings with silver contents similar to those of the present nanocomposites [43, 44]. Silver concentration in nanocomposites, as determined by EDS, was 5.1, 7.7, 10.4 and 24.5 wt% for RPS-AgNPs-125, RPS-AgNPs-250, RPS-AgNPs-500 and RPS-AgNPs-1000, respectively (Table 2). Showing a constant increment of Ag at.% in nanocomposites with immersion in higher Ag concentration solutions and indicating that AgNPs impregnation on RPS represents a good method to develop antibacterial nanocomposites with controlled concentrations of well-dispersed AgNPs. Preserving well-dispersed AgNPs upon impregnation on RPS is important because nanoparticles aggregation might significantly decrease the antibacterial activity of the RPS-AgNPs nanocomposites.

*Staphylococcus aureus* and *Stenotrophomonas maltophilia* isolated from burned patients (INR-16-1700 and HCR-392861) were multi-drug resistance bacteria. Nevertheless, both strains were affected by RPS-AgNPs nanocomposites, which significantly inhibited growth of *S. aureus* and *S. maltophilia* in agar and biofilm formation. Inhibition was observed from low AgNPs concentrations, where RPS-AgNPS-125 significantly inhibited *S. aureus* and *S. maltophilia* growth in agar culture plates in comparison to RPS. Nanocomposites showed bactericidal effects against Gram-positive and Gram-negative clinical multi-drug resistance bacteria, which might be convenient to overcome the problem of multi-drug resistance bacteria strains of clinical interest, and to provide broad-spectrum antibacterial protection, which could be quite useful to prevent polymicrobial colonization, as it is generally the case of infected burn lesions [45]. Moreover, AgNPS-RPS nanocomposites exhibited significant antibiofilm properties in comparison to RPS. Thus, results from microbial susceptibility in microdilution plate (Table 1), Kirby Bauer assay (Fig. 4 and Table 3) and colony biofilm model (Fig. 5) confirmed, together, the potential of RPS-AgNPs nanocomposites to be used as dressings to prevent microbial colonization of skin wounds. Cellular therapy using MSC is an excellent option to improve wound healing; however, it is necessary to have an appropriate way to deliver and keep the cells on the wound beds. Adipose tissue is a good option to isolate mesenchymal stem cells with therapeutic potential (Fig. 6) [46], because, even in burned patients, it could be possible to obtain healthy adipose tissue to isolate MSC for development of autologous cellular skin dressings. One advantageous avenue to achieve carrying of ADMSC to wound beds is the use of appropriate materials that work simultaneously as cell carriers to deliver cells to the wound and scaffolds to provide structural support for skin regeneration. RPS-AgNPs nanocomposites represent these materials plus the advantage of having antibacterial properties that will keep wounds free of infection. Nevertheless, previous works have shown that Ag nanoparticles can exert cytotoxic effects on eukaryote cells in a concentration dependent way [47]. In the present work, it was showed that antibacterial RPS-AgNPs-125 and RPS-AgNPs-250 nanocomposites were favorable for culture of adipose-derived mesenchymal stem cells, showing that cell viability was ≥ 80%; Fig. 7. Results indicated that the capability of nanocomposites to sustain cell culture significantly decreased as AgNPs concentration increased. Nevertheless, cells on RPS-AgNPs-125 and RPS-AgNPs-250 positively proliferate with culture days. The number of ADMSC on RPS-AgNPs-250 and RPS-AgNPs-500 at 24 h of culture was the smallest and cells were not able to proliferate. ADMSC viability percentages on RPS-AgNPS-125 and RPS-AgNPs-250 are very encouraging for the use of these two nanocomposites as cellular wound covers with skin regenerative potential. Another concern when using nanocomposites involving AgNPs is the potential cytotoxicity of silver products (AgNPs and Ag ions) released from nanocomposites over time. AgNPs-RPS nanocomposites developed in the present study showed that, upon incubation in water, initial silver release corresponded to ≈ 0.7 to 13 ppm for AgNPs-RPS-125 to AgNPs-RPS-1000, respectively, from 3.14 cm^2^ nanocomposite samples in 1 mL of water (Fig. 3). Cell viability (indirectly measured as the amount of metabolically active ADMSC) after 24 h of exposure to silver products released from nanocomposites was similar to that of CTL, which corresponded to cells incubated with fresh complemented DMEM. After 2 and 3 days of exposure to silver release products from AgNPS-RPS-125 and AgNPs-RPS250, cell viability was 77–61% in comparison to CTL; following the half maximal inhibitory concentration (IC50) criteria it is possible to say that silver release products from these two nanocomposites were not significantly cytotoxic against ADMSC [48]. On the contrary, silver release products from AgNPs-RPS-500 and AgNPS-RPS-1000 were significantly cytotoxic over time decreasing ADMSC viability to less than 50% in comparison to CTL from day 2 of exposure. Previous studies have reported average eukaryote cell viability ≈ 60–70% after 24 h of incubation with AgNPs of size between 8 and 75 nm. For some cells such as preosteoblasts MC3T3-E1 or adrenal medullar cells PC12 viability reported after 72 h of culture in presence of 9 nm nanoparticles is ≈ 60%, and significant cell viability decrements are reported after 72 h of culture with 46 nm nanoparticles [41]. This suggests that those nanoparticles might be less favorable for cell culture and proliferation than the present AgNPs used to develop RPS-AgNPS125 and RPS-AgNPs 250 nanocomposites; which allowed ADMSC viability and proliferation. Other studies have reported even lower cell viability (46% at 24 h of incubation) for nanocomposites with smaller contents of Ag (0.01% w/w) [49] than the ones of nanocomposites studied in the present work.

RPS-AgNPs nanocomposites were cytotoxic for eukaryote and prokaryote cells in a concentration-dependent manner. However, we were able to determine appropriate concentrations of AgNPs to develop RPS-AgNPs nanocomposites with the potential to simultaneously function as (1) antimicrobial covers with antibiofilm and bacteriostatic properties and (2) dressings with skin regenerative properties due to their capability to sustain mesenchymal stem cells culture. Results indicate that RPS-AgNPs nanocomposites could positively impact in the treatment of severe skin wounds such as diabetic ulcers or second-deep degree burns.

## Conclusions

Here it was showed that RPS-AgNPs nanocomposites with low Ag concentrations, developed by impregnation of silver nanoparticles on radiosterilized pig skin, inhibited the growth of bacteria and prevented biofilm formation; but at the same time, allowed mesenchymal stem cells culture. RPS-AgNPs-125 and RPS-AgNPs-250 nanocomposites seeded with ADMSC represent potential antibacterial cellular dressings to treat severe skin wounds, because impregnated AgNPs can prevent bacterial infection, RPS can provide cover to control massive loss of water and a cellular-matrix like scaffold to transport cells to the wound bed, and ADMSC can secrete growth factors to enhance wound healing.

## Additional file

**Additional file 1: Figure S1.** Schematic representation of gallic acid oxidation.

## Abbreviations

MSC: mesenchymal stem cells
ADMSC: adipose-derived mesenchymal stem cells
AgNPs: silver nanoparticles
RPS: radioesterilized pig skin
