# Supplementary figures and images for: Silver-pig skin nanocomposites and mesenchymal stem cells: suitable antibiofilm cellular dressings for wound healing
Source: J Nanobiotechnology. 2018 Jan 10;16:2. doi: 10.1186/s12951-017-0331-0 (PMC5761131; doi:10.1186/s12951-017-0331-0)

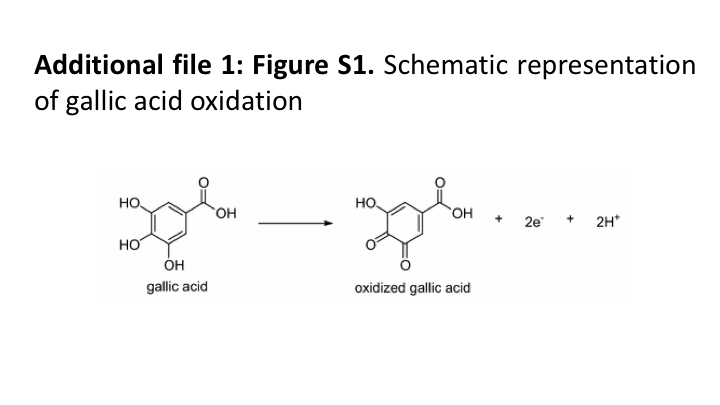

Supplement: Supplementary file 1 — Additional file 1: Figure S1. Schematic representation of gallic acid oxidation. [file 12951_2017_331_MOESM1_ESM.tiff]
